# Improving Lambda Red Genome Engineering in *Escherichia coli* via Rational Removal of Endogenous Nucleases

**DOI:** 10.1371/journal.pone.0044638

**Published:** 2012-09-05

**Authors:** Joshua A. Mosberg, Christopher J. Gregg, Marc J. Lajoie, Harris H. Wang, George M. Church

**Affiliations:** 1 Department of Genetics, Harvard Medical School, Boston, Massachusetts, United States of America; 2 Program in Chemical Biology, Harvard University, Cambridge, Massachusetts, United States of America; 3 Department of Systems Biology, Harvard Medical School, Boston, Massachusetts, United States of America; 4 Wyss Institute for Biologically Inspired Engineering, Harvard University, Boston, Massachusetts, United States of America; Saint Louis University, United States of America

## Abstract

Lambda Red recombineering is a powerful technique for making targeted genetic changes in bacteria. However, many applications are limited by the frequency of recombination. Previous studies have suggested that endogenous nucleases may hinder recombination by degrading the exogenous DNA used for recombineering. In this work, we identify ExoVII as a nuclease which degrades the ends of single-stranded DNA (ssDNA) oligonucleotides and double-stranded DNA (dsDNA) cassettes. Removing this nuclease improves both recombination frequency and the inheritance of mutations at the 3′ ends of ssDNA and dsDNA. Extending this approach, we show that removing a set of five exonucleases (RecJ, ExoI, ExoVII, ExoX, and Lambda Exo) substantially improves the performance of co-selection multiplex automatable genome engineering (CoS-MAGE). In a given round of CoS-MAGE with ten ssDNA oligonucleotides, the five nuclease knockout strain has on average 46% more alleles converted per clone, 200% more clones with five or more allele conversions, and 35% fewer clones without any allele conversions. Finally, we use these nuclease knockout strains to investigate and clarify the effects of oligonucleotide phosphorothioation on recombination frequency. The results described in this work provide further mechanistic insight into recombineering, and substantially improve recombineering performance.

## Introduction

Lambda Red recombination (“recombineering”) has emerged as a useful tool in genetics and molecular biology, facilitating the precise generation of insertions, deletions, and point mutations at loci specified by flanking homology regions of as little as 35 bp [1]. Lambda Red, as well as the similar RecET system [2], is capable of modifying *Escherichia coli* chromosomal [2], [3], plasmid [2], [4], and BAC [5], [6], [7], [8] targets using either dsDNA cassettes [2], [3] or ssDNA oligonucleotides [9], [10]. Both strategies have been used toward a broad array of powerful applications. Recombineering with dsDNA cassettes has been used to insert heterologous genes [11] and pathways [12] onto the *E. coli* chromosome for use in metabolic engineering and other efforts. Additionally, dsDNA recombineering has been applied to facilitate cloning and subcloning [13], [14], and to replace endogenous genes [15], enabling the creation of a library of single-gene knockout *E. coli* strains [16] and the generation of a strain of *E. coli* with 15% of its genome removed [17]. Recent applications of oligonucleotide recombineering have leveraged the ability of Lambda Red to simultaneously recombine multiple oligos (*i.e.* “Multiplex Automatable Genome Engineering”, or “MAGE”) [18]. This technique has been used to diversify and rapidly optimize the pathway coding for the biosynthesis of the small molecule lycopene [18], to engineer promoters [19], and to change all *E. coli* amber (TAG) stop codons into ochre (TAA) stop codons [20].

However, despite the versatility and power of Lambda Red recombineering, many applications are limited by the frequency of recombination. The low recombination frequency of gene insertion (∼5 × 10^−4^ recombinants per viable cell) [3] necessitates the linkage of inserted genes to selectable markers, preventing one-step, markerless gene insertion and the simultaneous insertion of multiple cassettes. Similarly, only a limited number of simultaneous mutations can reliably be generated in a given cycle of MAGE [20], thus constraining the degree to which a genome can conveniently be reengineered. Emerging mechanistic knowledge of Lambda Red recombination may identify strategies for overcoming these barriers.

Recently, it has been shown that the mechanism for Lambda Red dsDNA recombination proceeds by a similar process as oligonucleotide recombination [21], [22]. Three phage Lambda-derived proteins are required to mediate dsDNA recombination. The first, Gam, inhibits the endogenous RecBCD and SbcCD nucleases [23], which would otherwise degrade the exogenous dsDNA recombineering cassettes. The second protein, Lambda Exonuclease (Exo), degrades one of the two strands in its entirety, leaving behind a full-length strand of ssDNA [21], [22]. The third protein, Beta, then binds to this ssDNA and catalyzes its annealing to the lagging strand of the replication fork, where it is incorporated into the newly synthesized strand as part of an Okazaki fragment [21], [22]. Given this mechanism, recombination predominantly occurs when Lambda Exo degrades the leading-targeting strand of dsDNA, leaving behind the lagging-targeting strand for recombination at the replication fork. The final step of Beta-catalyzed annealing and incorporation as an Okazaki fragment is analogous to the accepted mechanism for Red-mediated oligonucleotide recombination, which requires only the Beta recombinase [24].

Mechanistic insight into Lambda Red recombination has already elucidated several means of improving recombination frequency. For instance, clarifying the mechanism of dsDNA recombination led to the insight that using phosphorothioate bonds to protect the lagging-targeting dsDNA strand would improve recombination frequency, while protecting the leading-targeting strand would not [21], [22]. Similarly, singleplex oligonucleotide recombination frequency was optimized to ∼30% from initial frequencies of ∼0.2% by targeting oligos to the lagging strand of the replication fork [25], removing the mismatch repair protein MutS [26] or evading its action via modified bases [27], minimizing internal oligo secondary structure [18], optimizing homology lengths [18], and introducing phosphorothioate bonds to prevent nuclease degradation of oligonucleotides [18]. Recently, the frequency of multiplex oligonucleotide recombination has also been greatly improved by directing an oligo to repair a mutated antibiotic resistance gene in the vicinity of the loci targeted by the other recombineering oligos [28]. Thus, co-selection MAGE (CoS-MAGE) enriches for cells with high levels of recombination near the corrected selectable marker, and increases recombination frequencies significantly.

In this work, we focus on removing endogenous nuclease enzymes as a means of further improving recombineering properties. Many lines of evidence suggest that endogenous nucleases limit recombination. As discussed previously, using phosphorothioate bonds to protect oligonucleotides [18] and dsDNA cassettes [21], [22] has been shown to improve recombination frequency, suggesting that endogenous nuclease degradation can render these cassettes non-recombinogenic. This is bolstered by the recent observation that knocking out four potent ssDNA exonucleases improves singleplex oligonucleotide recombination frequency when low concentrations of oligos are used [29]. Furthermore, it has been shown that mutations located near the ends of an oligonucleotide [27] or dsDNA cassette [21] are inherited less often than mutations located closer to the interior of the oligo or cassette. This further implies degradation of oligonucleotides and dsDNA, and suggests that this nuclease processing prevents the inheritance of mutations along the full length of a cassette. We reasoned that by inactivating certain endogenous nucleases, we could improve recombination frequency and the preservation of mutations encoded at the ends of cassettes. In this work, we first investigate the nuclease processing of dsDNA, and identify ExoVII as a key nuclease which degrades the ends of dsDNA cassettes. Removing this nuclease facilitates preservation of mutations on the ends of dsDNA cassettes as well as ssDNA oligos. This discovery led us to investigate nuclease processing in the context of MAGE, and to identify a set of nucleases capable of degrading recombineering oligonucleotides. Removal of these nucleases provides for substantial improvement of recombination frequency, thereby facilitating greater diversity generation and the creation of more simultaneous mutations.

## Results

### Using Phosphorothioated Cassettes to Investigate Nuclease Processing of dsDNA

**Figure 1:**
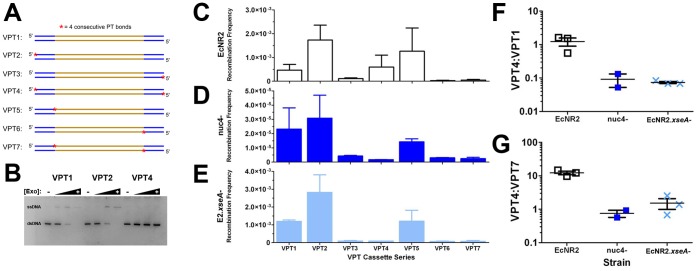
Investigating Nuclease Processing with Variably Phosphorothioated Cassettes. A) Diagram of the variably phosphorothioated (VPT) cassette series. Homology arms to *lacZ* are shown in blue, and the inserted heterologous *kanR* gene is shown in gold; the lagging-targeting strand is represented as the top strand, and the leading-targeting strand is represented as the bottom strand. The red asterisk indicates the presence of 4 consecutive phosphorotiohate bonds. **B)** *In vitro* Lambda Exo digestion of non-phosphorothioated (VPT1, left), single end phosphorothioated (VPT2, center), and dually phosphorothioated (VPT4, right) dsDNA cassettes. Samples were digested with no enzyme (lanes 1, 5, & 9) and tenfold-increasing amounts of enzyme, from left to right. The bottom band is dsDNA, and the top band is the ssDNA product after Lambda Exo degrades one of the two strands. PAGE analysis confirms that dually phosphorothioated cassettes are protected from degradation. **C–E)** Recombination frequencies for the VPT cassette series in EcNR2 (C), nuc4^−^ (D), and EcNR2.*xseA* ^−^ (E). Insertion frequencies were measured as the number of kanamycin resistant recombinants over the total number of cfu (as plated on non-selective media). These data are presented as the mean with the error bars as standard deviation; n = 3 for EcNR2 and EcNR2.*xseA* ^−^, and n = 2 for nuc4^−^. Numerical data are shown in Table S2. **F& G)** Cassette insertion frequencies were measured in technical replicates for EcNR2, nuc4^−^, and EcNR2.*xseA* ^−^. Data in F) & G) are presented as the mean with the standard error of the mean. The ratio between VPT4 and VPT1 (F) indicates a strain’s ability to recombine cassettes with terminal PT bonds preventing the direct action of Lambda Exo. The ratio between VPT4 and VPT7 (G) indicates whether a strain is more able to process terminal PT bonds (VPT4) in comparison with internal PT bonds (VPT7).

Our observations regarding the recombination frequencies of phosphorothioated dsDNA cassettes [21] led us to investigate the possibility that the ends of cassettes are routinely degraded by endogenous nucleases. Phosphorothioate (PT) bonds have been reported to block Lambda Exo from degrading dsDNA cassettes [30], an observation that we have confirmed in an *in vitro* experiment using recombinant Lambda Exo (Figure 1A–B). Despite this, placing PT bonds on both 5′ ends of a dsDNA cassette does not decrease (and actually enhances) the recombination frequency of that cassette *in vivo* [21], [22]. This is surprising, given that according to the current mechanism, Lambda Exo must bind to a 5′ dsDNA end and degrade that strand in order for recombination to occur [21], [22]. Prior mechanisms for Red-mediated dsDNA recombination [24], [31] have similar implications, requiring Lambda Exo to degrade both 5′ ends of a dsDNA cassette. It is not immediately apparent how a cassette with both 5′ ends phosphorothioated could be processed by Lambda Exo and undergo recombination. It is possible that Lambda Exo behaves differently *in vivo* than *in vitro* (e.g. due to the presence of protein partners or cofactors), and is able to degrade phosphorothioate bonds in a cellular context. Alternatively, an endogenous *E. coli* nuclease may degrade the 5′ PT bonds on one or both strands, thereby allowing Lambda Exo to degrade the remainder of the strand and generate the ssDNA recombination intermediate.

To investigate this, we generated a set of seven variably phosphorothioated (VPT) *lacZ::kanR* dsDNA insertion cassettes (Figure 1A) with four PT bonds located either terminally, at the 5′ end of the cassette, or internally, between the homology and heterology regions. These PT bonds were placed on neither strand, on the lagging-targeting strand only, on the leading-targeting strand only, or on both strands. These constructs were recombined into EcNR2 (strain genotypes given in Table 1), and recombination frequencies were calculated by dividing the number of kanamycin-resistant recombinant cfu (colony forming units) by the number of total cfu. Results are shown in Figure 1C and in Table S2.

**Table 1:** Genotypes of Abbreviated Strains.

Strain Name	Strain Genotype
EcNR2	*Escherichia coli* MG1655 Δ*mutS*::*cat* Δ(*ybhB*-*bioAB*)::[λcI857 N(*cro-ea59*)::*tetR*-*bla*]
nuc4^−^	EcNR2.*xonA* ^−^,*recJ* ^−^,*xseA* ^−^,*exoX* ^−^
nuc5^−^	EcNR2.*xonA* ^−^,*recJ* ^−^,*xseA* ^−^,*exoX* ^−^,*redα* ^−^

Genotypes of all abbreviated strains are presented here for clarity.

Several trends are apparent. Relative to unmodified dsDNA (VPT1), terminal PT bonds are beneficial when placed on the lagging-targeting strand (VPT2), as they protect the recombinogenic strand from being degraded. Conversely, terminal PT bonds are slightly detrimental when located on the leading-targeting strand (VPT3), as they interfere with the ability of Lambda Exo to degrade that strand and generate the lagging-targeting ssDNA intermediate. The cassette with terminal PTs placed on both strands (VPT4) is roughly equivalent (1.23 times more recombinogenic, as shown in Figure 1F) to unmodified dsDNA (VPT1), presumably due to a cancellation of the two effects discussed above.

Internally located PT bonds, however, show a much more striking effect. When on the lagging-targeting strand only (VPT5), they do not decrease recombination frequency. However, internally located PT bonds on the leading-targeting strand (VPT6 and VPT7) are highly detrimental to recombination frequency. These cassettes have substantially lower recombination frequencies than the corresponding terminally phosphorothioated cassettes (VPT3 and VPT4, respectively), with a VPT4:VPT7 ratio of 12.3 (Figure 1G) and a VPT3:VPT6 ratio of 4.9. This suggests that internally located PT bonds are more effective than terminal PT bonds at blocking Lambda Exo from degrading the leading-targeting strand and generating the ssDNA recombination intermediate. Thus, it is unlikely that Lambda Exo can independently degrade phosphorothioates *in vivo*; rather, it appears that an endogenous nuclease is able to cleave terminally located PT bonds, thereby allowing Lambda Exo to degrade the remainder of the strand. Internally located PT bonds are not directly accessible to this endogenous nuclease, and/or cannot be removed without rendering the cassette non-recombinogenic due to the degradation of one of its homology regions. Thus, blocking the leading-targeting strand with internal PT bonds has a uniquely detrimental effect on recombination frequency.

These results suggest a strategy for identifying the nuclease(s) responsible for degrading the phosphorothioated ends of dsDNA cassettes: inactivate candidate nucleases, and then compare the recombination frequencies of unmodified dsDNA (VPT1) and dsDNA with terminal PT bonds on both strands (VPT4). If the nuclease(s) responsible for pruning terminal PT bonds are still present, then VPT4 will have a recombination frequency that is roughly equal to that of VPT1 (VPT4:VPT1 ≈ 1.0, as in EcNR2). However, if the nuclease(s) have been removed, the strain will no longer be able to degrade the phosphorothioate bonds on the ends of the VPT4 dsDNA. Thus, the recombination frequency of VPT4 will drop well below that of VPT1 (VPT4:VPT1<<1), as Lambda Exo can no longer generate the ssDNA recombination intermediate.

We first tested VPT1 and VPT4 in a strain lacking Endonuclease I (*endA*), a potent periplasmic endonuclease capable of degrading dsDNA [32]. This resulted in a VPT4:VPT1 ratio of 1.55, similar to that observed for the EcNR2 strain (Table 2). Thus, Endonuclease I is not responsible for degrading the phosphorothioated ends of dsDNA cassettes.

**Table 2:** Identifying the Nuclease(s) which Degrade Phosphorothioated Cassette Ends.

Strain Background	VPT4:VPT1
EcNR2.*xonA* ^−^,*recJ* ^−^,*exoX* ^−^ (*xseA* ^+^)	**0.77**
EcNR2.*xonA* ^−^,*xseA* ^−^,*exoX* ^−^ (*recJ* ^+^)	**0.06**
EcNR2.*recJ* ^−^,*xseA* ^−^,*exoX* ^−^ (*xonA* ^+^)	**0.09**
EcNR2.*xonA* ^−^,*recJ* ^−^,*xseA* ^−^ (*exoX* ^+^)	**N/A**
EcNR2	**1.23**
EcNR2.*endA* ^−^	**1.55**
EcNR2.*xonA* ^−^,*recJ* ^−^,*xseA* ^−^,*exoX* ^−^ (nuc4^−^)	**0.09**
EcNR2.*xseA* ^−^	**0.07**

In order to determine which of the nucleases removed from nuc4^−^ is responsible for cleaving terminal PT bonds from dsDNA, four different strains were created with only three of the four nuc4^−^ nucleases inactivated. VPT1 and VPT4 cassettes were recombined into all strains, and the ratio of number of recombinants calculated. Only the strain containing functional *xseA* had nearly as many VPT4 recombinants as VPT1 recombinants. This suggests that *xseA* (ExoVII) is the nuclease responsible for cleaving PT bonds off the end(s) of VPT4, thereby rendering the cassette recombinogenic. EcNR2.*xonA* ^−^,*recJ* ^−^,*xseA* ^−^ did not give kanR recombinants for either cassette; this strain was recreated by restoring *exoX* function in nuc4^−^, whereupon it exhibited the same non-recombinogenic phenotype. The biological basis of this phenotype is unclear. VPT4:VPT1 ratios for EcNR2, EcNR2.*endA* ^−^, nuc4^−^, and EcNR2.*xseA* ^−^ are also shown for comparison.

We next tested a strain which lacked the four primary [33] *E. coli* ssDNA exonucleases – *recJ*/RecJ, [34] *xonA*/ExoI, [35] *xseA*/ExoVII, [36] and *exoX*/ExoX [37]. This strain (EcNR2.*recJ* ^−^,*xonA* ^−^,*xseA* ^−^,*exoX* ^−^, or “nuc4^−^”; Table 1) demonstrated a VPT4:VPT1 ratio of 0.09, far lower than that of EcNR2 (Table 2). Thus, recombination frequency data for the full VPT series was collected in this strain (Figure 1D). These data show a strikingly different trend than that observed for EcNR2. The recombination frequency of VPT4 is indeed far lower than that of VPT1 (Figure 1F). Additionally, terminal PT bonds on the leading-targeting strand are just as detrimental to recombination frequency as internal PT bonds (VPT3:VPT6 = 1.41; VPT4:VPT7 = 0.75, Figure 1G). Because constructs with terminal PT bonds on the leading-targeting strand are minimally recombinogenic in nuc4^−^, this suggests that these constructs can no longer be processed by Lambda Exo, and therefore that the nuclease(s) capable of cleaving off these PT bonds is/are absent in this strain. Residual recombinants for such cassettes may be due to a limited ability of Lambda Exo to degrade PT bonds, or to slight nuclease activity that is still present.

To identify which nuclease(s) is/are responsible for cleaving terminal PT bonds, four strains were generated, each of which restored one of the four nucleases removed in nuc4^−^ (*i.e.* three of the four nucleases were inactivated in these strains). VPT1 and VPT4 were recombined into these strains, and the VPT4:VPT1 ratio of recombinants was measured (Table 2). One of the four strains (EcNR2.*recJ* ^−^,*xonA* ^−^,*xseA* ^−^) was surprisingly found to be non-recombinogenic. This was the case regardless of whether the strain was generated by inactivating the three nucleases from an EcNR2 background, or by reactivating exoX in nuc4^−^; this suggests that the non-recombinogenic phenotype did not arise from an off-target mutation. Of the remaining strains, two showed a VPT4:VPT1 ratio similar to that of nuc4^−^, while the strain containing *xseA* (EcNR2.*recJ* ^−^,*xonA* ^−^,*exoX* ^−^) showed a ratio similar to that of EcNR2. This suggests that ExoVII (*xseA*) may be the nuclease responsible for the ability of EcNR2 to degrade phosphorothioated ends of dsDNA cassettes. An ExoVII single knockout strain (EcNR2.*xseA* ^−^) was therefore generated.

### Recombineering Properties of the ExoVII Mutant Strain

The VPT series of cassettes was recombined into EcNR2.*xseA* ^−^, and recombination frequencies were calculated (Figure 1E). This strain shows a pattern of relative frequencies similar to that of nuc4^−^: terminal phosphorothioation on the leading-targeting strand is highly detrimental to recombination frequency, and has impact equivalent to that of internal phosphorothioation (Figure 1F–G). This suggests that these terminal PT bonds can no longer be cleaved off, and therefore that ExoVII is the primary *E. coli* nuclease responsible for degrading phosphorothioated ends of dsDNA cassettes. Moreover, removing ExoVII significantly increases the recombination frequency of unmodified dsDNA (VPT1) above levels observed in EcNR2 (p = 0.0076), suggesting that the action of ExoVII on the ends of dsDNA cassettes may compromise recombination frequency.

ExoVII is a nuclease encoded by the *xseA* and *xseB* genes [38]. It is processive, and can degrade from either the 5′ or 3′ end [39]. While it is highly specific for ssDNA substrates, it is capable of degrading short overhangs and then continuing into duplex regions of DNA [39]. Its observed ability to degrade dsDNA ends is likely due to strand “breathing,” possibly aided by endogenous helicase enzymes, or by PT bonds lowering the annealing temperature between the two strands [40]. Alternatively, another endogenous 3′-to-5′ exonuclease may degrade part of the strand complementary to the 5′ PT bonds, leaving behind an ssDNA overhang to which ExoVII can bind. While ExoVII is classified as an exonuclease due to its requirement for a free ssDNA end, it technically has endonucleolytic activity, given that its degradation products are 4–12 bp oligonucleotides [39]. Thus, ExoVII likely cleaves to the 3′ end of the four 5′ terminal PT bonds used in the VPT cassette series, rather than degrading the PT bonds directly. This explains the ability of ExoVII to readily process terminally phosphorothioated cassettes.

**Figure 2:**
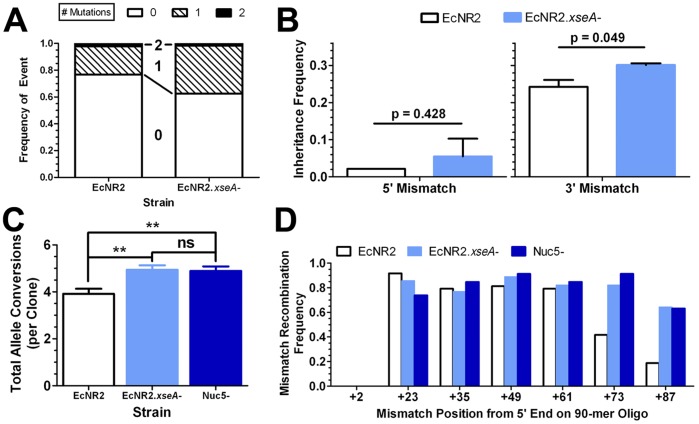
Removal of ExoVII Preserves Mutation Inheritance. A) A non-phosphorothioated *lacZ::kanR* dsDNA cassette with two 2 bp sets of mismatch mutations (placed 7&8 bp from each end of the cassette) was recombined into EcNR2 and EcNR2.*xseA* ^−^ cells. Resulting kanR recombinants were then genotyped in order to determine whether they inherited these distally-located mutations. The distribution of mutations in EcNR2 (two independent experiments, n = 180) and EcNR2.*xseA* ^−^ (two independent experiments, n = 177) was plotted as the frequency of clones inheriting 0 (empty black bars), 1 (hatched black bars), or 2 (filled black bars) sets of mutations. **B)** When the inheritance of each 2 bp mutation is considered individually, both show increased preservation in EcNR2.*xseA* ^−^, although this is only statistically significant for the 2 bp mutation on the 3′ end of the cassette (as defined with respect to the lagging-targeting strand). Data are presented as the mean with the standard deviation from the mean. **C & D)** A 90mer lagging strand-targeting oligonucleotide was designed to introduce 7 stop codons (placed at the +2, +23, +35, +49, +61, +73, +87 base pair positions with respect to the 5′ end) into the *lacZ* gene. This oligo was transformed into EcNR2, EcNR2.*xseA* ^−^, and nuc5^−^ cells. LacZ^−^ cells were identified and their *lacZ* genes sequenced in order to determine which mutations were conferred in a given clone. The average total number of stop codons introduced into a given clone is shown in C). Both EcNR2.*xseA* ^−^ (**p = 0.0004, n = 54) and nuc5^−^ (**p = 0.001, n = 46) exhibit similar, statistically significant increases in total mutation inheritance compared to EcNR2 controls (n = 45). A breakdown of each strain’s inheritance of each mutation is given in D). Both EcNR2.*xseA* ^−^ and nuc5^−^ show similarly increased inheritance of 3′-located mutations, but no increased inheritance of 5′-located mutations. No clones inherited the mutation encoded at position +2. Data in C) are presented as the mean with the standard error of the mean.

Given that the action of ExoVII on the ends of dsDNA cassettes appears to compromise recombination frequency, we investigated whether its removal would increase the inheritance of mutations encoded near the ends of the homology regions of a dsDNA insertion cassette. Thus, we designed a non-phosphorothioated *lacZ::kanR* cassette with 2 bp mutations encoded 7 and 8 bp from both ends of the cassette. This construct was then recombined into EcNR2 and EcNR2.*xseA* ^−^. KanR colonies were selected, and their genotypes were analyzed at the mutation loci (Figure 2A–B). The strain with ExoVII removed shows greater inheritance of these end-located mutations (Figure 2A), although the difference is only statistically significant for the mutation encoded at the 3′ end of the cassette (as defined with respect to the lagging-targeting strand; Figure 2B, right panel). This confirms that ExoVII also degrades non-phosphorothioated dsDNA cassettes, and that it is partially responsible for the poor inheritance of mutations located at the ends of such cassettes. However, given that the EcNR2.*xseA* ^−^ strain still gives only low levels of inheritance of these mutations, it is likely that other endogenous nucleases are also responsible for degrading the ends of dsDNA cassettes.

Because ExoVII is an ssDNA exonuclease [39], we next set out to determine whether its removal has an ability to facilitate the inheritance of end-located mutations in oligonucleotides as well as dsDNA cassettes. We tested a 90mer oligonucleotide previously designed to disrupt the *lacZ* gene [27], with seven premature stop codons distributed along its sequence. This oligo was protected with four PT bonds on each end. This “lacZ.7.stop” oligo was recombined into three strains – EcNR2, EcNR2.*xseA* ^−^, and nuc5^−^ (EcNR2.*recJ*, ^−^ *xonA* ^−^,*xseA* ^−^,*exoX* ^−^,*redα* ^−^; Table 1), a strain with Lambda Exo removed along with all four of the potent ssDNA exonucleases. LacZ^−^ colonies were identified by IPTG/X-Gal screening, and *lacZ* was amplified from these clones for sequencing analysis. From these sequences, it was determined which of the seven mutations were inherited in a given clone, and the proportion of recombinants with each mutation was thereby calculated.

The results shown in Figure 2C–D suggest that nuc5^−^ and EcNR2.*xseA* ^−^ facilitate the inheritance of end-located mutations. Nuc5^−^ clones exhibited significantly more of the mutations encoded by the lacZ.7.stop oligo (4.94±0.20, **p = 0.001, Figure 2C) compared to EcNR2 (3.92±0.22). This was due to enhanced inheritance of mutations at the 3′ end of the 90mer oligonucleotide (Figure 2D). Interestingly, removing ExoVII phenocopies the performance of nuc5^−^, leading to improved mean conversions (4.89±0.18, **p = 0.004, Figure 2C) compared to EcNR2, and no significant difference from nuc5^−^ (p = 0.8416). Moreover, EcNR2.*xseA* ^−^ also provides for significantly greater inheritance of mutations at the 3′ end of the 90mer oligonucleotide (Figure 2D). Despite the dual polarity of ExoVII, its removal has no apparent effect on the inheritance of 5′ mutations, nor does the removal of the other four exonucleases in nuc5^−^. This suggests that another unidentified nuclease may be responsible for degrading the 5′ ends of oligonucleotides. Such degradation may be occurring postsynaptically, possibly mediated by the 5′-to-3′ exonuclease domain of polymerase I [41]. The 3′ protection effect of the ExoVII knockout strain is equivalent to that observed for the nuc5^−^ strain, suggesting that ExoVII is the only one of the five removed exonucleases which compromises the inheritance of mutations along the full length of phosphorothioated oligos.

### Nuclease Knockouts Improve MAGE Performance

Given that ExoVII degrades oligonucleotides and may hinder their ability to recombine, we investigated whether the removal of ExoVII could improve oligonucleotide recombination frequency. It has previously been shown [29] that removing the four potent *E. coli* ssDNA exonucleases improves singleplex recombination frequency, but only when low concentrations of oligo are used. Since oligonucleotide concentration can easily be increased, this has little practical benefit. However, nuclease removal may provide a greater benefit when multiple oligonucleotides are recombined simultaneously, as in MAGE [18]. Previous results [28] have shown that the recombination frequency of a given oligo is directly proportional to the mole fraction of that oligo in a complex mixture, even when the oligo is present at concentrations that would be saturating for singleplex oligo recombination. We hypothesize that this apparent competition between oligonucleotides is due to a limited number of oligos entering each cell during electroporation. Thus, if several oligos are simultaneously co-electroporated, the resulting intracellular concentration of any given oligo will be low. Presynaptic nuclease degradation may therefore have a large negative impact on recombination frequency in MAGE.

To investigate this, we compared the MAGE performance of EcNR2 and EcNR2.*xseA* ^−^. The nuc5^−^ strain discussed previously was also tested in order to determine if other exonucleases impact MAGE performance. Lambda Exo was removed from this strain along with the four potent ssDNA exonucleases, as Lambda Exo has been shown [42] to have trace activity against ssDNA, and is not required for oligo recombination. CoS-MAGE [28] was used in these experiments, so as to determine whether the nuclease knockout strains are able to improve upon the current best practices for MAGE. Three different sets of 10 recombineering oligos (each specifying a single TAG→TAA mutation, as designed previously) [20] were tested, so as to ascertain the MAGE performance of the strains at multiple loci, and in both replichores. This was done in order to confirm the robustness of the results, as oligo recombination frequency can vary due to largely unelucidated oligo-specific and locus-specific effects [20]. Each of the three oligo sets was paired with a co-selection oligo which restored the function of a nearby mutated antibiotic resistance gene, thereby selecting for high recombination frequency in the vicinity of the targeted loci. All recombineering oligos had two PT bonds on each end, as was previously optimized for MAGE [20]. Targeted loci were screened by mascPCR [20] to determine which alleles were converted in a given clone.

**Figure 3:**
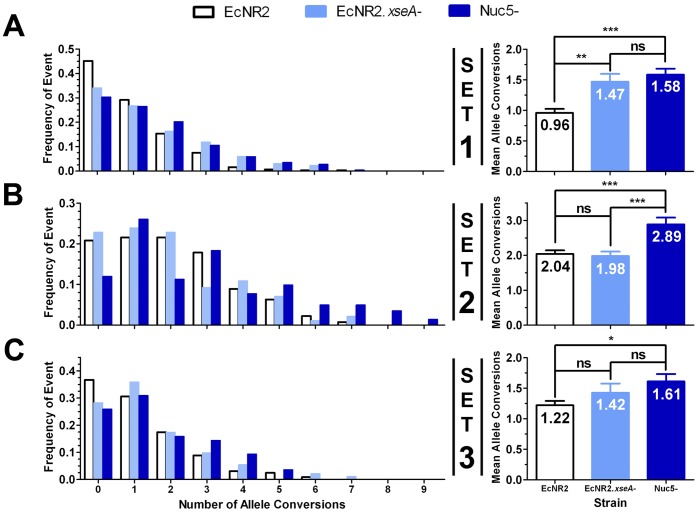
Effect of Nuclease Genotype on CoS-MAGE Performance. CoS-MAGE was carried out in three strains (EcNR2, EcNR2.*xseA* ^−^, and nuc5^−^), using sets of ten oligos encoding TAGTAA mutations, and a co-selection oligo designed to revert a mutated selectable marker located within 500 kb of the targeted loci. Set 1 (**A**) was co-selected with chloramphenicol acetyltransferase (*cat,* inserted at the *mutS* locus). In comparison with EcNR2 (n = 319), both EcNR2.*xseA* ^−^ (**p = 0.0001, n = 135) and nuc5^−^ (***p<0.0001, n = 257) show statistically significant increases in mean oligo conversion, a decreased proportion of clones exhibiting no allele conversions, and more clones with 5+ conversions. Set 2 (**B**) was co-selected with beta lactamase (*bla,* inserted with the λ prophage). Here, nuc5^−^ (n = 142) shows a statistically significant increase in recombineering performance compared to both EcNR2 (***p<0.0001, n = 268) and EcNR2.*xseA* ^−^ (***p<0.0001, n = 184). Set 3 (**C**) was co-selected with *tolC*. Here, nuc5^−^ (n = 139) shows a statistically significant increase in mean allele conversion compared to EcNR2 (*p = 0.002, n = 327). EcNR2.*xseA* ^−^ (n = 92) shows an intermediate phenotype between EcNR2 (p = 0.2) and nuc5- (p = 0.3). All oligos used in this experiment had two PT bonds on both ends. Data shown in the right panels are presented as the mean with the standard error of the mean. Statistical significance is denoted using a starred system where ns denotes a non-significant variation, * denotes p<0.003, ** denotes p<0.001, and *** denotes p<0.0001.

Results are shown in Figure 3. For all three recombineering oligo sets (Sets 1–3 in Figure 3A–C, respectively), nuc5^−^ significantly outperforms EcNR2 (***p<0.0001, ***p<0.0001, *p = 0.002, respectively). An average of 46% more alleles are converted per clone in nuc5^−^, and the frequency of clones with 5 or more conversions is increased by 200%. Furthermore, nuc5^−^ reduces the frequency of clones with no conversions by 35%. All of these improvements are particularly important given that MAGE can be performed in iterative cycles, thereby compounding these recombination enhancements. Thus, removing all potent ssDNA exonucleases significantly improves the performance of CoS-MAGE.

The EcNR2.*xseA* ^−^ strain appears to have properties intermediate between those of EcNR2 and nuc5^−^. Although EcNR2.*xseA* ^−^ exhibits a statistically significant increase in MAGE performance with Set 1 (1.47±0.13) compared to EcNR2 (0.96±0.07, **p = 0.0001), this strain’s performance with Sets 2 & 3 was not statistically different from EcNR2 (p = 0.7 & 0.2, respectively). Given that Set 1 exhibited the largest difference in performance between EcNR2 and nuc5^−^ (65% higher allele conversion in nuc5^−^), it is possible that Set 1 is the most susceptible to nuclease repression, and therefore that the effect of removing ExoVII would be most apparent for this set. Overall, using these three tested sets, nuc5^−^ is superior to EcNR2.*xseA* ^−^. This suggests that the action of ExoVII somewhat compromises CoS-MAGE frequency, but that some or all of the other exonucleases removed in nuc5^−^ also have a role in oligo degradation.

### Examining the Effect of Phosphorothioate Bonds on MAGE Frequency

**Figure 4:**
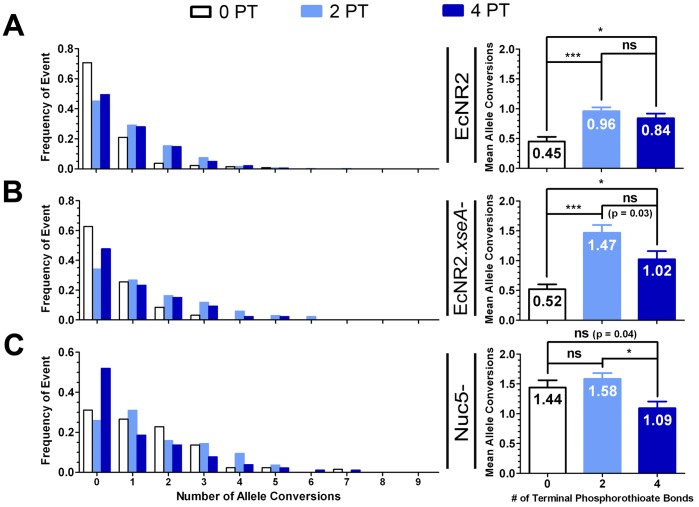
Effect of Phosphorothioate Bonds on CoS-MAGE Performance. The effect of oligo phosphorothioation on the CoS-MAGE performance of the various nuclease knockout strains was studied. Variants of recombineering oligo Set 1 (described in Figure 3) with no PT bonds (0 PT) and four PT bonds on both ends (4 PT) were compared with the initial version of Set 1, which had two PT bonds on both ends (2 PT). Recombination was performed with *cat* co-selection as prior. For EcNR2 (**A**), n = 133 (0 PT), n = 319 (2 PT), n = 186 (4 PT). For EcNR2.*xseA* ^−^ (**B**), n = 94 (0 PT), n = 92 (2 PT), n = 86 (4 PT). For nuc5^−^ (**C**), n = 132 (0 PT), n = 257 (2 PT), n = 183 (4 PT). Data shown in the right panels are presented as the mean with the standard error of the mean. Statistical significance is denoted using a starred system where ns denotes a non-significant variation, * denotes p<0.003, ** denotes p<0.001, and *** denotes p<0.0001.

As noted, the above experiments were performed with recombineering oligonucleotides with two PT bonds on each end. We next sought to determine whether the optimal number of PT bonds was the same for each of our strains, whether the benefits of nuc5^−^ could be recapitulated simply by adding more PT bonds to the recombineering oligos, and whether the differences between the strains would be more pronounced if no PT bonds were used. Thus, we recombined EcNR2 (Figure 4A), EcNR2.*xseA* ^−^ (Figure 4B), and nuc5^−^ (Figure 4C) with versions of recombineering oligo Set 1, co-selected with a restoration oligo for the nearby *cat* gene. A non-phosphorothioated version of oligo Set 1 was tested, as was a version with 4 PT bonds on each end. The resulting allele conversion distributions were determined as above, and compared with those previously observed for the version of Set 1 with 2 PT bonds on each end.

The results are shown in Figure 4. Notably, for all tested strains, the oligo set with 2 PT bonds gave the greatest average number of allele conversions per clone. Comparatively, 4 PT bonds had a detrimental effect on recombination frequency that was most notable in nuc5^−^ (Figure 4C), where the 0 PT set (1.44±0.12) outperformed the 4 PT set (1.09±0.11, p = 0.04). Similarly, for EcNR2.*xseA* ^−^ (Figure 4B), the 2 PT set (1.47±0.13) exhibited a significant local optimum compared to the 0 PT set (0.52±0.08, ***p<0.0001) and the 4 PT set (1.02±0.14, *p = 0.002), corroborating the detrimental effect of too many PT bonds. Thus, the EcNR2.*xseA* ^−^ and nuc5^−^ strains confer recombineering advantages that cannot be recapitulated simply by preventing nuclease degradation via the use of more PT bonds. The detrimental effect of high levels of phosphorothioation may be due to PT bonds decreasing the strength of the annealing interaction between the oligo and the lagging strand of the replication fork [40]. Alternatively, oligos with PT bonds may be somewhat toxic, thereby killing cells that take up a large number of oligos and would otherwise yield many converted alleles.

The relative performance of the differently phosphorothioated oligo sets in the various strains suggests that this detrimental effect of PT bonds is countervailed by the beneficial effect of nuclease protection. In EcNR2 (Figure 4A), which has a full complement of endogenous nucleases, the 4 PT set (0.84±0.08) greatly outperforms the 0 PT set (0.45±0.08, **p = 0.0006), suggesting that the effect of nuclease protection outweighs the detrimental impact of the PT bonds on recombination frequency. Conversely, in nuc5^−^ (Figure 4C), where nuclease degradation is mitigated by the nuclease knockouts, the 0 PT set (1.44±0.12) slightly outperforms the 4 PT set (1.09±0.11, p = 0.04), suggesting that the detrimental impact of the PT bonds outweighs any beneficial effect of nuclease protection. In this strain, the 2 PT set (1.58±0.10) is statistically equivalent to the 0 PT set (1.44±0.12, p = 0.38), suggesting that most or all relevant nuclease activity has been abrogated. However, some level of residual exonuclease activity in nuc5^−^ is suggested by the strain’s poor inheritance of mutations encoded at the 5′ end of oligos (Figure 2D).

When no PT bonds are used to protect the recombineering oligonucleotides, the nuc5^−^ strain yields roughly threefold more allele conversions per average clone than EcNR2. This stands to reason, as non-PT oligos are likely to be particularly susceptible to the activity of endogenous ssDNA exonucleases. Similarly, EcNR2.*xseA* ^−^ also has low recombination frequency (roughly similar to that of EcNR2) when 0 PT oligos are used. This suggests that the exonucleases other than ExoVII (*i.e.* RecJ, Exo I, ExoX, and Lambda Exo) are readily capable of degrading non-protected oligos. However, when PT bonds are used, EcNR2.*xseA* ^−^ notably outperforms EcNR2, and is only slightly less recombinogenic than nuc5^−^ for this oligo set. In sum, these results reinforce the importance of using phosphorothioated oligos when performing MAGE in a strain containing endogenous nucleases, but caution that the overuse of phosphorothioates can be detrimental.

## Discussion

The results described in this paper provide additional insight into the cellular processes occurring during recombineering. First, the discovery that ExoVII degrades the ends of dsDNA cassettes solves the mystery of why dsDNA with both 5′ ends phosphorothioated is still recombinogenic despite PT bonds blocking Lambda Exo. Dually-phosphorothioated dsDNA enters the cell, after which ExoVII degrades the 5′ PT bonds of one or both strands. This step may also require the action of a helicase or another endogenous nuclease in order to generate ssDNA ends to which ExoVII can bind. After the action of ExoVII, Lambda Exo degrades the rest of the leading-targeting strand, leaving behind the lagging-targeting ssDNA recombination intermediate [21], [22]. Therefore, dually-phosphorothioated dsDNA recombines with a frequency equal to or exceeding that of unmodified dsDNA despite requiring the action of one or more endogenous nucleases in addition to Lambda Exo. This suggests that there is a great deal of interaction between endogenous nucleases and recombineering cassettes. The variable dsDNA recombination frequencies exhibited in this study by nuclease knockout strains support this notion. For example, the nuc4^−^ strain exhibits decreased recombination frequency for all tested VPT cassettes. The origin of this phenotype is uncertain; it is possible that one of the removed exonucleases has a role in dsDNA recombination, or that the presence of exogenous linear dsDNA is somewhat toxic to cells with these four nucleases removed, thereby selecting against cells which take up the cassettes necessary for recombination. Along similar lines, strain EcNR2.*recJ* ^−^,*xonA* ^−^,*xseA* ^−^ appears to be non-recombinogenic, for reasons that are unclear, while EcNR2.*xseA* ^−^ has slightly enhanced recombination frequency for cassettes without PT bonds blocking the leading-targeting strand.. Taken together, this suggests that modifying endogenous nuclease activity may be a powerful lever for affecting Lambda Red recombination, and possibly a valuable strategy for engineering further improvements of dsDNA recombination frequency.

The VPT series recombination frequency data for EcNR2 (Figure 1C) also further supports the recently proposed dsDNA recombineering mechanism involving a full-length ssDNA intermediate [21], [22]. Other proposed mechanisms [24], [31] have suggested an intermediate in which the 5′ ends of both homology regions have been recessed by Lambda Exo, leaving behind the dsDNA heterology region flanked by 3′ homology ssDNA overhangs. These proposed mechanisms have different implications on the expected recombination frequencies of certain VPT series cassettes. According to the recently proposed mechanism, VPT7 would be expected to have a recombination frequency significantly less than that of unmodified dsDNA, as PT bonds between the 5′ homology and heterology regions of both strands would prevent Lambda Exo from degrading one strand and generating a full-length ssDNA intermediate. In contrast, the placement of PT bonds in VPT7 would be expected to facilitate Lambda Exo’s generation of the 3′ overhang intermediate suggested in the other proposed mechanisms. Thus, if this mechanism were correct, VPT7 would be expected to have equal or greater recombination frequency than unmodified dsDNA. As shown in Figure 1C and Table S2, VPT7 has 10.2-fold lower recombination frequency than unmodified dsDNA (VPT1) in EcNR2. This result is consistent with those from similar previous experiments [22], [43], supporting the proposed mechanism involving a full-length ssDNA intermediate, and further refuting the previously proposed mechanisms.

This work also lends more insight into recombineering with oligonucleotides. We have shown that removing selected endogenous nucleases improves multiplex recombination frequency, despite the use of a high total concentration (5.2 µM) of oligos. This suggests that the concentration of any given oligo within the cell is not enough to overcome the action of endogenous nucleases, and therefore that oligo entry into the cell is a limiting factor in MAGE. This observation initially appears to contradict the conclusion presented in a recent study by Sawitzke *et al*. [29], which showed that the recombination frequency of a given oligonucleotide could be increased by the addition of non-specific carrier oligos which prevent endogenous exonucleases from degrading the recombinogenic oligo. However, this phenomenon was tested only for very low concentrations of the recombinogenic oligo (up to 0.01 µM). Even at this concentration, adding carrier oligos (at 0.1 µM) had less than a 2-fold enhancement of recombination frequency; a more pronounced enhancement was observed only for very low concentrations of recombinogenic oligo (0.001 µM and below). In concentration regimes typical for MAGE (>1 µM total oligos), it has been shown that adding a second oligonucleotide *decreases* the recombination frequency of the first oligonucleotide [28]. Presumably, at these concentrations (which have been previously been shown to be optimal for recombination frequency [18] and were therefore used in this work) any benefit conferred by the saturation of endogenous exonucleases is outweighed by competition for cellular entry. These findings therefore suggest that enhancing oligo uptake is likely to be a fruitful avenue for further improving MAGE.

Additionally, studying the consequences of different levels of oligo phosphorothioation in the EcNR2, EcNR2.*xseA* ^−^ and nuc5^−^ strains enabled the deconvolution of the countervailing effects of phosphorothioation. Phosphorothioate bonds improve recombination frequency by protecting oligos against nuclease degradation, but also hinder recombination frequency – possibly by reducing the strength of the annealing interaction between the oligo and the lagging strand of the replication fork, or by causing toxicity to the cell. Two PT bonds on both ends of recombineering oligonucleotides was found to be optimal for all three strains tested in this work, but future nuclease knockout strains will need to be optimized in order to determine the ideal number of PT bonds for oligos recombined into that strain.

Beyond providing additional mechanistic insight into Lambda Red recombination, this work also achieves several important improvements of oligo and dsDNA recombineering. Firstly, removing ExoVII was shown to improve the inheritance of mutations at the 3′ ends of oligonucleotides and dsDNA cassettes. This may be particularly useful in oligonucleotide recombineering, as it allows more mutations to be reliably introduced by a single oligo. This could be leveraged for several applications, such as simultaneously modifying several residues near the active site of a protein, recoding [20] a larger region with a given oligonucleotide, or modifying several genetic features (e.g. promoter strength, ribosome binding site strength, and the presence or absence of a premature stop codon) with a single oligo. Similarly, the improvement of MAGE recombination frequency via the use of EcNR2.*xseA* ^−^ and nuc5^−^ will also have substantial utility. These strains facilitate greater modification of a population of cells in a single MAGE cycle (or in multiple cycles), which will be useful for projects that seek the rapid allelic diversification of a population of cells [18]. Such advancements are also expected to be useful for improving the Red-mediated diversification of BACs and plasmids. Similarly, the enhanced recombination frequency of these strains also means that fewer cycles will be needed in order to achieve an isogenic recoded population of cells, or to identify a strain with all desired genetic changes among a set of screened clones.

Finally, this work provides guidelines as to the appropriate strain to use for a given recombineering application. It should be noted that the nuc5^−^ strain was observed to have poor regrowth after electroporation, taking roughly twice as long as EcNR2 or EcNR2.*xseA* ^−^ to recover to confluence. The pre-electroporation growth rate of nuc5^−^ was only slightly less than those of EcNR2 and EcNR2.*xseA* ^−^ (150 minutes *vs.* 125 minutes to reach mid-log growth phase from a 1∶100 dilution of overnight culture), likely due to the ability of *mutS* removal to suppress the known cold-sensitive growth phenotype of *recJ*/*xonA*/*xseA*/*exoX* quadruple mutants [44]. Therefore, while nuc5^−^ has somewhat better multiplex recombination frequency than EcNR2.*xseA* ^−^, its poor regrowth properties cause each recombination cycle to take notably longer. Thus, using EcNR2.*xseA* ^−^ is likely optimal for applications in which multiple cycles are necessary; the nuc5^−^ strain may be preferable for applications in which a single cycle is sufficient and fast regrowth is not necessary. While quadruple mutants for *recJ, xonA, xseA,* and *exoX* have increased point mutation rates, this phenomenon is epistatic to *mutS* [44]. Given that strains used for genome engineering are often *mutS* ^−^, removing these nucleases (as in nuc5^−^) does not further exacerbate the mutator phenotype. However, the combined removal of *xonA*, *recJ*, and *exoX* has been shown to increase rates of rearrangement mutations involving repetitive sequences [45], and therefore this strain should not be used for applications in which genomic stability is paramount. Directed evolution and/or the restoration of selected nucleases may facilitate improved growth rates and genomic stability, without substantially compromising recombination frequency. This may be a valuable avenue for future study.

In conclusion, this work helps elucidate how endogenous nucleases act on ssDNA and dsDNA cassettes used for recombineering. In addition to providing further mechanistic insight into Lambda Red recombination, this work also facilitated the creation of nuclease knockout strains with markedly improved recombination frequency and mutation inheritance. These strains will be highly useful as chassis in future recombineering efforts, and may enable new and powerful applications of Lambda Red technology.

## Materials and Methods

A full list of primers and recombineering oligonucleotides used in this work is presented in Table S1. All oligonucleotides were ordered from Integrated DNA Technologies with standard purification and desalting.

### Generating dsDNA Recombineering Cassettes

Both the VPT series of dsDNA recombineering cassettes described above and the *lacZ::kanR* cassette with mutations encoded in its homology arms were generated by PCR using the primers denoted in Table S1. PCRs were performed using Kapa HiFi HotStart ReadyMix, with primer concentrations of 0.5 µM, and 0.1 ng of *lacZ::kanR* dsDNA (generated in previous work [21]) used as template. PCRs had a total volume of 50 µL, and were heat activated at 95°C for 5 min, then cycled 30 times with a denaturation temperature of 98°C (20 sec), an annealing temperature of 62°C (15 sec), and an extension temperature of 72°C (45 sec). PCRs were brought to 72°C for 5 min, and then held at 4°C. PCR products were cleaned with the Qiagen PCR purification kit (elution in 50 µL H_2_O). Samples were desalted with Microcon® Ultracel YM-100 columns (spinning twice at 500 × g for 20 min, and bringing to 500 µL in H_2_O before each spin). Resulting samples were then brought to 100 µL in H_2_O and quantitated on a NanoDrop™ ND1000 spectrophotometer. All samples were analyzed on a 1% agarose/EtBr gel to confirm that the expected band was present and pure.

### *In vitro* dsDNA Digestion by Lambda Exo

*LacZ::kanR* dsDNA (20 ng) with zero, one, or both ends phosphorothioated (VPT1, VPT2, and VPT4, respectively; see Figure 1A) was added to 9 µL of 1X Lambda Exonuclease Buffer (New England Biolabs). Lambda Exonuclease (New England Biolabs) was serially diluted in 1X Lambda Exonuclease Buffer as needed and 1 µL was then added to the reaction. Reactions were incubated at 37°C for 30 min, heat inactivated at 75°C for 10 min, and then analyzed on an Invitrogen 6% TBE non-denaturing PAGE gel (180 V, 40 min, post-stained in Invitrogen SYBR Gold for 15 min).

### Strain Creation

EcNR2 (*Escherichia coli* MG1655 Δ*mutS*::*cat* Δ(*ybhB*-*bioAB*)::[λcI857 N(*cro-ea59*)::*tetR*-*bla*]) [18] was used as the basis for all strains created in this work. From an EcNR2 background, nuclease genes *endA, xonA*, *recJ*, *xseA*, *exoX*, and *redα* (Lambda Exo) were inactivated singly or in combination (as described in the Results section) via oligo-mediated Lambda Red recombination as described below. Knockout oligos (Table S1) were designed to introduce a premature stop codon and a frameshift mutation at the beginning of the nuclease gene, thereby rendering the targeted nuclease inactive. Strains used for CoS-MAGE were generated by recombining an oligonucleotide designed to inactivate a chromosomal resistance marker (*cat*, *tolC*, or *bla*) and identifying resulting colonies with the appropriate sensitivity to antibiotic or SDS [46].

### Performing Lambda Red Recombination

Lambda Red recombinations with ssDNA and dsDNA were performed as previously described [18], [21]. In brief, cultures were grown in LB Lennox media (10 g tryptone, 5 g yeast extract, 5 g NaCl per 1 L water), from a 1∶100 dilution of an overnight culture. Cultures were placed in a rotator drum at 30°C until they reached an OD_600_ of 0.4–0.6 (typically 2.25–2.5 hrs). Lambda Red expression was then induced by shaking cultures at 300 rpm in a 42°C water bath (15 min). Induced cultures were immediately cooled on ice, and 1 mL of cells were washed twice in ice cold deionized water (dH_2_O). The cell pellet was resuspended in 50 µL of dH_2_O containing the intended recombineering construct(s). For experiments in which dsDNA cassettes were recombined, 100 ng was used. For experiments in which a single oligonucleotide was recombined, 1 µM of oligo was used. For experiments in which sets of ten recombineering oligos were recombined along with a co-selection oligo, 0.5 µM of each recombineering oligo was used, along with 0.2 µM of the co-selection oligo (5.2 µM total). Samples were electroporated (BioRad GenePulser™, 0.1 cm cuvette, 1.78 kV, 200 Ω, 25 µF), and allowed to recover in 3 mL LB Lennox in a rotator drum at 30°C. Cultures were recovered for at least 3 hours (5+ hours in CoS-MAGE experiments, so as to eliminate polyclonal colonies), and then plated and analyzed as below.

### Analyzing Recombination

In order to analyze the recombination of *lacZ::kanR* insertion cassettes, recovery cultures were plated onto LB Lennox plates with kanamycin sulfate (30 µg/mL), incubated at 30°C overnight, and the number of resulting colonies counted. To determine the total number of cells present, recovery cultures were diluted and plated onto LB Lennox plates with carbenicillin (50 µg/mL). These results were used to determine the recombination frequency of a given sample (# recombinants/# total cells), or the ratio of the number of kanR recombinants yielded by two different cassettes in a given strain. All platings were performed in duplicate.

Allele-specific colony PCR (ascPCR) or multiplex allele-specific colony PCR (mascPCR) [20] was used to detect the 1–2 bp mutations generated in the CoS-MAGE experiments, the inactivation of endogenous nucleases, and the recombination of the *lacZ::kanR* cassette with mutations encoded in its homology arms. In these experiments, two PCRs are performed for each tested clone – one with a forward primer designed to hybridize to the wild type sequence at a locus targeted by a recombineering cassette, and one with a forward primer designed to hybridize to the mutated sequence conferred by the recombineering cassette; the same reverse primer is used in both reactions. If the mutant-detecting PCR gives a band but the wild type detecting PCR does not, the clone is scored as a recombinant. In mascPCR, primer sets for interrogating several wild type or mutant loci are combined in a single reaction, and each amplicon has a different size ranging from 100 bp to 850 bp. Singleplex ascPCR reactions (used to determine whether a given nuclease had been inactivated) were performed with Kapa 2GFast HotStart ReadyMix including 10X Kapa dye. PCR reactions had a total volume of 20 µL, with 0.5 µM of each primer, and a template of 1 µL of stationary phase culture derived from a given clone. These PCRs were carried out with an initial activation step at 95°C for 2 min, then cycled 30 times with a denaturation temperature of 95°C (15 sec), an annealing temperature of 63–67°C (15 sec; temperature as optimized for a given pair of ascPCR reactions), an extension temperature of 72°C (40 sec), and a final extension at 72°C for 90 sec.

CoS-MAGE recovery cultures were plated on media selective for the co-selected resistance marker (LB Lennox with 50 µg/mL carbenicillin for *bla*, 20 µg/mL chloramphenicol for *cat*, or 0.005% SDS for *tolC*, with 20 µg/mL chloramphenicol added to enhance the robustness of selection), and targeted loci in the resulting clones were screened by mascPCR using Kapa 2GFast Multiplex PCR ReadyMix with 10X Kapa dye. PCRs to detect the two mutations encoded in the homology arms of the *lacZ::kanR* cassette were also performed in multiplex, as were PCRs to simultaneously genotype several nuclease knockout mutations. Monoclonal colonies were grown to stationary phase under proper antibiotic selection, and PCR template was prepared by diluting 2 uL of culture into 100 uL of dH_2_O. These mascPCR reactions had a total volume of 10 µL, with 0.2 µM of each primer and 2 µL of template. These PCRs were carried out with an initial activation step at 95°C for 3 min, then cycled 26 times with a denaturation temperature of 95°C (15 sec), an annealing temperature of 63–67°C (30 sec; temperature as optimized for a given pair of mascPCR reactions), an extension temperature of 72°C (1 min), and a final extension at 72°C for 5 min. All ascPCRs and mascPCRs were analyzed on 1.5% agarose/EtBr gels (180 V, 60 min).

We repeated recombination experiments with the mutation-encoding *lacZ::kanR* cassette twice for EcNR2 and EcNR2.*xseA* ^−^. In each experiment, 96 individual colonies were genotyped for each strain. Only monoclonal and unambiguous mascPCR results were counted towards the final analysis presented here. 5′ and 3′ mutation inheritance data points were each combined and analyzed for statistically significant differences between strains using the Mann-Whitney U-test with significance defined as p<0.05.

In CoS-MAGE experiments, all strains were recombined with all oligo sets at least twice. Replicates were combined to generate a complete data set for each strain’s performance with each set of oligos. At least 96 colonies were genotyped for each strain tested with each recombineering oligo set. Only monoclonal and unambiguous mascPCR results were counted toward the final analysis presented here. Given the large sample sizes tested here (n >85), we used parametric one way ANOVA to test for significant variance in the CoS-MAGE performance of the strains (EcNR2, EcNR2.*xseA* ^−^, nuc5^−^) for a given oligo set. Subsequently, we used a Student’s t-test to make pairwise comparisons with significance defined as p<0.05/n, where n is the number of pairwise comparisons. Here, n = 15, as these data were planned and collected as part of a larger set with 6 different strains, although only the results for EcNR2, EcNR2.*xseA* ^−^, and nuc5^−^ are presented here. As such, significance was defined as p<0.003 for the analyses presented in Figures 3 & 4. Statistical significance in Figures 3 & 4 are denoted using a system where * denotes p<0.003, ** denotes p<0.001, and *** denotes p<0.0001. For the experiment in which oligo sets were tested with 0, 2, or 4 PTs on both ends, comparisons were made between EcNR2, EcNR2.*xseA* ^−^, and nuc5^−^ (the only three tested strains in this experiment) for each of the three differently phosphorothioated oligo sets, separately. Additionally, comparisons were made between each oligo set for each of the three strains, also separately. Thus, 15 pairwise comparisons were performed, and significance thresholds are as above.

For the experiment in which a *lacZ*-inactivating oligo with 7 stop codons (lacZ.7.stop) was recombined, recombinants were identified as white colonies on plates containing Fisher ChromoMax™ IPTG/X-Gal solution, and recombination frequencies (# of white colonies/total # of colonies) were determined for every replicate. For the white colonies, the entire *lacZ* open reading frame was amplified with primers lacZ.seq-f and lacZ.seq-2-r (Table S1), using Kapa HiFi HotStart ReadyMix and the same conditions as described above for this enzyme. Samples were then purified with the Qiagen PCR purification kit, also as described above, and quantitated on a NanoDrop™ ND1000 spectrophotometer. Purified DNA was submitted to Genewiz for Sanger sequencing (40 ng DNA, with 25 pmol of either lacZ.seq-f or lacZ.seq-2-r primer). Good quality sequence pairs were stitched together using SeqMan (Lasergene DNAstar) and exported as FASTA sequences. These sequences were then analyzed for their genotype at the loci where the lacZ.7.stop oligo could impart inheritance of mutations. The recombinations and recombination frequency counts were repeated thrice so as to ensure consistency, but sequencing was only performed on one biological replicate. Mean allele conversion metrics were generated from the sequencing data by scoring each mutation locus as 1 for a mutant sequence or 0 for a wild type sequence. We tested for statistically significant variance in mean allele conversions using a parametric one way ANOVA. Subsequently, we used a Student’s t-test to make pairwise comparisons with significance defined as p<0.05/3, *i.e.* p<0.01. Statistical significance in Figure 2C is denoted using a starred system where * denotes p<0.01, ** denotes p<0.001, and *** denotes p<0.0001.

## Supporting Information

Table S1 **Oligonucleotides Used in this Study.**
(PDF)

Table S2 **Recombination Frequencies of the VPT Cassette Series in Nuclease Knockout Strains.**
(PDF)
