# Supplementary material for: Improving Lambda Red Genome Engineering in Escherichia coli via Rational Removal of Endogenous Nucleases
Source: PLoS One. 2012 Sep 5;7(9):e44638. doi: 10.1371/journal.pone.0044638 (PMC3434165; doi:10.1371/journal.pone.0044638)
Supplement: Table S1 — Oligonucleotides Used in this Study. (PDF) [file pone.0044638.s001.pdf]

Table S1. Oligonucleotides Used in this Study

All oligonucleotides were ordered from Integrated DNA Technologies with standard purification and desalting. An asterisk in the sequence represents a phosphorothioate bond. Oligos from the TAG→TAA recombineering sets are denoted "X.Y", where X is the set that the oligo is from, and Y is the size of the mascPCR band corresponding to that locus. For mascPCR primers, "wt" denotes a primer designed to amplify the wild type sequence at a targeted locus, "mut" denotes a primer designed to amplify the mutated sequence conferred by a recombineering oligo, and "rev" denotes the reverse primer for amplifying a targeted locus. For oligo sequences denoted with a §, variants with no PT bonds and 4 PT bonds at each end were also used.

Oligo Name:	Used for:	Sequence:
ygaR	Set 1.850 TAG-->TAA	§G*C*GAAGATCAGTAAAGATATAGAAGGTGGTATCCCTGGCTATTAACAAGGTCAGGTTTTGATTCCATTCATTAAAGATCCAGTAACAA*A*A
yqaC	Set 1.700 TAG-->TAA	§A*T*TAAAAATTATGATGGGTCCACGCGTGTCGGCGGTGAGGCGTAACTTAATAAAGGTTGCTCTACCTATCAGCAGCTCTACAATGAAT*T*C
gabT	Set 1.600 TAG-->TAA	§T*C*ACCATTGAAGACGCTCAGATCCGTCAGGGTCTGGAGATCATCAGCCAGTGTTTTGATGAGGCGAAGCAGTAACGCCGCTCCTATGC*C*G
ygaU	Set 1.500 TAG-->TAA	§T*G*ACGCCAATTCCCATTATCCAGCAGGCGATGGCTGGCAATTAATTACTCTTCCGGAATACGCAACACTTGCCCCGGATAAATTTTAT*C*C
ygaM	Set 1.400 TAG-->TAA	§G*T*AGGTATTTTTATCGGCGCACTGTTAAGCATGCGCAAATCGTAATGCAAAAATGATAATAAATACGCGTCTTTGACCCCGAAGCCTG*T*C
luxS	Set 1.300 TAG-->TAA	§T*T*TGAACTGGCTTTTTTCAATTAATTGTGAAGATAGTTTACTGATTAGATGTGCAGTTCCTGCAACTTCTCTTTCGGCAGTGCCAGTT*C*T
mltB	Set 1.250 TAG-->TAA	§A*A*TTTTACGAGGAGGATTCAGAAAAAAGCTGATTAGCCAGAGGGAAGCTCACGCCCCCCTCTTGTAAATAGTTACTGTACTCGCGCCA*G*C
srlE	Set 1.200 TAG-->TAA	§A*C*TGTACTGATCGCCTGGTTTGTCTCCGGTTTTATCTATCAATAAAGGCTGAAACATGACCGTTATTTATCAGACCACCATCACCCGT*A*T
norW	Set 1.150 TAG-->TAA	§A*T*CGGATGAAAGAGGCATTTGGATTGTTGAAAACATTGCCGATGTAAGTGGGCTACTGTGCCTAAAATGTCGGATGCGACGCTGGCGC*G*T
ascB	Set 1.100 TAG-->TAA	§A*T*CATTCTGGTGGTATAAAAAAGTGATTGCCAGTAATGGGGAAGATTTAGAGTAAGTAACAGTGCCGGATGCGGCGTGAACGCCTTAT*C*C
bioD	Set 2.850 TAG-->TAA	T*C*GAAGACGCGATCTCGCTCGCAATTTAACCAAATACAGAATGGTTACAACAAGGCAAGGTTTATGTACTTTCCGGTTGCCGCATTTT*C*T
moaE	Set 2.700 TAG-->TAA	C*G*TAAACGTATGTACTGAGCGGTGAAATTGCCGGACGCAGCGGTGCCTTATCCGGCTAACAAAAAATTACCAGCGTTTTGCCGCCTGC*T*G

ybhM	Set 2.600 TAG-->TAA	G*C*GATGTGAAGTTTAGTTAAGTTCTTTAGTATGTGCA<br>TTTACGGTTAATGAAAAAAACGCGTATGCCTTTGCCAG<br>ACAAGCGTTATAG*C*T
ybhS	Set 2.500 TAG-->TAA	T*T*TATCGGCCTGACGTGGCTGAAAACCAAACGTCGG<br>CTGGATTAAGGAGAAGAGCATGTTTCATCGCTTATGG<br>ACGTTAATCCGCAAA*G*A
ybiH	Set 2.400 TAG-->TAA	C*A*TATCGACCTGATTTTGCAAGGATTATCGCAAAGG<br>AGTTTGTAATGATGAAAAAACCTGTCGTGATCGGATTG<br>GCGGTAGTGGTACT*T*G
ybiR	Set 2.300 TAG-->TAA	T*C*TGAATTAATCTTCAAAACTTAAAGCAAAAGGCGGA<br>CTCATAATCCGCCTTTTTTATTTGCCAGACCTTAGTTG<br>GCCGGGAGTATAA*C*T
yliD	Set 2.250 TAG-->TAA	T*T*TCCTGTGAGGTGATTACCCTTTCAAGCAATATTCA<br>AACGTAATTATCCTTTAATTTTCGGATCCAGCGCATCG<br>CGTAAACCATCGC*C*C
yliE	Set 2.200 TAG-->TAA	G*A*CTGACTGTAAGTACGAACTTATTGATTCTGGACAT<br>ACGTAAATTACTCTTTTACTAATTTTCCACTTTTATCCC<br>AGGCGGAGAATG*G*C
ybjK	Set 2.150 TAG-->TAA	T*C*GGTTCAAGGTTGATGGGTTTTTTGTTATCTAAAAC<br>TTATCTATTACCCTGCAACCCTCTCAACCATCCTCAAA<br>ATCTCCTCGCGCG*A*T
rimK	Set 2.100 TAG-->TAA	C*G*CAAAAAGCGCAGGCAAAACCATGATCAGTAATGT<br>GATTGCGATTAACCACCCGTTTTCAGGCAATATTCTGT<br>CGTAGCGTGGCGTT*C*G
ygfJ	Set 3.850 TAG-->TAA	C*C*GGACGACTTTATTACAGCGAAGGAAAGGTATACT<br>GAAATTTAAAAAACGTAGTTAAACGATTGCGTTCAAAT<br>ATTTAATCCTTCCG*G*C
recJ	Set 3.700 TAG-->TAA	G*G*GATTGTACCCAATCCACGCTCTTTTTTATAGAGAA<br>GATGACGTTAAATTGGCCAGATATTGTCGATGATAATT<br>TGCAGGCTGCGGT*T*G
argO	Set 3.600 TAG-->TAA	C*T*CTGGAGGCAAGCTTAGCGCCTCTGTTTTATTTTTC<br>CATCAGATAGCGCTTAACTGAACAAGGCTTGTGCATG<br>AGCAATACCGTCTC*T*C
yggU	Set 3.500 TAG-->TAA	A*A*TCCGCAACAAATCCCGCCAGAAATCGCGGCGTTA<br>ATTAATTAAGTATCCTATGCAAAAAGTTGTCCTCGCAA<br>CCGGCAATGTCGGT*A*A
mutY	Set 3.400 TAG-->TAA	G*T*GGAGCGTTTGTTACAGCAGTTACGCACTGGCGC<br>GCCGGTTTAACGCGTGAGTCGATAAAGAGGATGATTT<br>ATGAGCAGAACGATTT*T*T
glcC	Set 3.300 TAG-->TAA	G*C*CACCATTTGATTCGCTCGGCGGTGCCGCTGGAG<br>ATGAACCTGAGTTAACTGGTATTAAATCTGCTTTTCAT<br>ACAATCGGTAACGCT*T*G
yghQ	Set 3.250 TAG-->TAA	A*C*TGAGTCAGCCGAGAAGAATTTCCCCGCTTATTCG<br>CACCTTCCTTAAATCAGGTCATACGCTTCGAGATACTT<br>AACGCCAAACACCA*G*C
yghT	Set 3.200 TAG-->TAA	T*G*GTTGATGCAGAAAAAGCGATTACGGATTTTATGA<br>CCGCGCGTGGTTATCACTAATCAAAAATGGAAATGCC<br>CGATCGCCAGGACCG*G*G
ygiZ	Set 3.150 TAG-->TAA	T*T*CTCTGTCTATGAGAGCCGTTAAAACGACTCTCATA<br>GATTTTATTAATAGCAAAATATAAACCGTCCCCAAAAA<br>AGCCACCAACCAC*A*A
yqiB	Set 3.100 TAG-->TAA	A*G*GGTTAACAGGCTTTCCAAATGGTGTCCTTAGGTT<br>TCACGACGTTAATAAACCGGAATCGCCATCGCTCCAT<br>GTGCTAAACAGTATC*G*C
ygaR_wt-f	Set 1.850_wt-f mascPCR	AAGGTGGTATCCCTGGCTATTAG

yqaC_wt-f	Set 1.700_wt-f mascPCR	CGGCGGTGAGGCGTAG
gabT_wt-f	Set 1.600_wt-f mascPCR	TTTTGATGAGGCGAAGCAGTAG
ygaU_wt-f	Set 1.500_wt-f mascPCR	GTTGCGTATTCCGGAAGAGTAG
ygaM_wt-f	Set 1.400_wt-f mascPCR	GTTAAGCATGCGCAAATCGTAG
luxS_wt-f	Set 1.300_wt-f mascPCR	GTTGCAGGAACTGCACATCTAG
mltB_wt-f	Set 1.250_wt-f mascPCR	GCTGGCGCGAGTACAGTAG
srlE_wt-f	Set 1.200_wt-f mascPCR	GGTTTGTCTCCGGTTTTATCTATCAATAG
norW_wt-f	Set 1.150_wt-f mascPCR	GATTGTTGAAAACATTGCCGATGTAG
ascB_wt-f	Set 1.100_wt-f mascPCR	CCAGTAATGGGGAAGATTTAGAGTAG
bioD_wt-f	Set 2.850_wt-f mascPCR	AGTACATAAACCTTGCCTTGTTGTAG
moaE_wt-f	Set 2.700_wt-f mascPCR	GCGGCAAAACGCTGGTAG
ybhM_wt-f	Set 2.600_wt-f mascPCR	AAGGCATACGCGTTTTTTTCATTAG
ybhS_wt-f	Set 2.500_wt-f mascPCR	CCAAACGTCGGCTGGATTAG
ybiH_wt-f	Set 2.400_wt-f mascPCR	AAGGATTATCGCAAAGGAGTTTGTAG
ybiR_wt-f	Set 2.300_wt-f mascPCR	TTAGTTATACTCCCGGCCAACTAG
yliD_wt-f	Set 2.250_wt-f mascPCR	CGCTGGATCCGAAAATTAAAGGATAG
yliE_wt-f	Set 2.200_wt-f mascPCR	TGGGATAAAAGTGGAAAATTAGTAAAAGAGTAG
ybjK_wt-f	Set 2.150_wt-f mascPCR	TTGAGAGGGTTGCAGGGTAG
rimK_wt-f	Set 2.100_wt-f mascPCR	GCCTGAAAACGGGTGGTTAG
ygfJ_wt-f	Set 3.850_wt-f mascPCR	AGCGAAGGAAAGGTATACTGAAATTTAG
recJ_wt-f	Set 3.700_wt-f mascPCR	TCATCGACAATATCTGGCCAATTTAG
argO_wt-f	Set 3.600_wt-f mascPCR	TGCACAAGCCTTGTTCAGTTAG
yggU_wt-f	Set 3.500_wt-f mascPCR	CAGAAATCGCGGCGTTAATTAATTAG
mutY_wt-f	Set 3.400_wt-f mascPCR	GGCGCGCCGGTTTAG
glcC_wt-f	Set 3.300_wt-f mascPCR	GCTGGAGATGAACCTGAGTTAG
yghQ_wt-f	Set 3.250_wt-f mascPCR	CTCGAAGCGTATGACCTGATTTAG
yghT_wt-f	Set 3.200_wt-f mascPCR	CGCGCGTGGTTATCACTAG
ygiZ_wt-f	Set 3.150_wt-f mascPCR	TGGGGACGGTTTATATTTTGCTATTAG
yqiB_wt-f	Set 3.100_wt-f mascPCR	CGATGGCGATTCCGGTTTATTAG
ygaR_mut-f	Set 1.850_mut-f mascPCR	AAGGTGGTATCCCTGGCTATTAA
yqaC_mut-f	Set 1.700_mut-f mascPCR	CGGCGGTGAGGCGTAA
gabT_mut-f	Set 1.600_mut-f mascPCR	TTTTGATGAGGCGAAGCAGTAA
ygaU_mut-f	Set 1.500_mut-f mascPCR	GTTGCGTATTCCGGAAGAGTAA
ygaM_mut-f	Set 1.400_mut-f mascPCR	GTTAAGCATGCGCAAATCGTAA
luxS_mut-f	Set 1.300_mut-f mascPCR	GTTGCAGGAACTGCACATCTAA
mltB_mut-f	Set 1.250_mut-f mascPCR	GCTGGCGCGAGTACAGTAA

srlE_mut-f	Set 1.200_mut-f mascPCR	GGTTTGTCTCCGGTTTTATCTATCAATAA
norW_mut-f	Set 1.150_mut-f mascPCR	GATTGTTGAAAACATTGCCGATGTAA
ascB_mut-f	Set 1.100_mut-f mascPCR	CCAGTAATGGGGAAGATTTAGAGTAA
bioD_mut-f	Set 2.850_mut-f mascPCR	AGTACATAAACCTTGCCTTGTTGTAA
moaE_mut-f	Set 2.700_mut-f mascPCR	GCGGCAAAACGCTGGTAA
ybhM_mut-f	Set 2.600_mut-f mascPCR	AAGGCATACGCGTTTTTTTCATTAA
ybhS_mut-f	Set 2.500_mut-f mascPCR	CCAAACGTCGGCTGGATTAA
ybiH_mut-f	Set 2.400_mut-f mascPCR	AAGGATTATCGCAAAGGAGTTTGTAA
ybiR_mut-f	Set 2.300_mut-f mascPCR	TTAGTTATACTCCCGGCCAACTAA
yliD_mut-f	Set 2.250_mut-f mascPCR	CGCTGGATCCGAAAATTAAAGGATAA
yliE_mut-f	Set 2.200_mut-f mascPCR	TGGGATAAAAGTGGAAAATTAGTAAAAGAGTAA
ybjK_mut-f	Set 2.150_mut-f mascPCR	TTGAGAGGGTTGCAGGGTAA
rimK_mut-f	Set 2.100_mut-f mascPCR	GCCTGAAAACGGGTGGTTAA
ygfJ_mut-f	Set 3.850_mut-f mascPCR	AGCGAAGGAAAGGTATACTGAAATTTAA
recJ_mut-f	Set 3.700_mut-f mascPCR	TCATCGACAATATCTGGCCAATTTAA
argO_mut-f	Set 3.600_mut-f mascPCR	TGCACAAGCCTTGTTCAGTTAA
yggU_mut-f	Set 3.500_mut-f mascPCR	CAGAAATCGCGGCGTTAATTAATTAA
mutY_mut-f	Set 3.400_mut-f mascPCR	GGCGCGCCGGTTTAA
glcC_mut-f	Set 3.300_mut-f mascPCR	GCTGGAGATGAACCTGAGTTAA
yghQ_mut-f	Set 3.250_mut-f mascPCR	CTCGAAGCGTATGACCTGATTTAA
yghT_mut-f	Set 3.200_mut-f mascPCR	CGCGCGTGGTTATCACTAA
ygiZ_mut-f	Set 3.150_mut-f mascPCR	TGGGGACGGTTTATATTTTGCTATTAA
yqiB_mut-f	Set 3.100_mut-f mascPCR	CGATGGCGATTCCGGTTTATTAA

ygaR_rev	Set 1.850_rev mascPCR	TAGGTAGAGCAACCTTTATTAAGCTACG
yqaC_rev	Set 1.700_rev mascPCR	TAAAAATATCTACATTTCTGAAAAATGCGCA
gabT_rev	Set 1.600_rev mascPCR	GCGGCGATGTTGGCTT
ygaU_rev	Set 1.500_rev mascPCR	AGGGTATCGGGTGGCG
ygaM_rev	Set 1.400_rev mascPCR	CGCAACGCTTCTGCCG
luxS_rev	Set 1.300_rev mascPCR	ATGCCCAGGCGATGTACA
mltB_rev	Set 1.250_rev mascPCR	AGACTCGGCAGTTGTTACGG
srlE_rev	Set 1.200_rev mascPCR	GGATGGAGTGCACCTTTCAAC
norW_rev	Set 1.150_rev mascPCR	GTGTTGCATTTGGACACCATTG
ascB_rev	Set 1.100_rev mascPCR	CGCTTATCGGGCCTTCATG
bioD_rev	Set 2.850_rev mascPCR	CGGGAAGAACTCTTTCATTTCGC
moaE_rev	Set 2.700_rev mascPCR	CGTCAATCCGACAAAGACAATCA
ybhM_rev	Set 2.600_rev mascPCR	TTACTGGCAGGGATTATCTTTACCG
ybhS_rev	Set 2.500_rev mascPCR	CTGTTGTTAGGTTTCGGTTTTCCT
ybiH_rev	Set 2.400_rev mascPCR	GTCATAGGCGGCTTGCG
ybiR_rev	Set 2.300_rev mascPCR	ATGAGCCGGTAAAAGCGAC
yliD_rev	Set 2.250_rev mascPCR	AATAAAATTATCAGCCTTATCTTTATCTTTTCGTATAAA
yliE_rev	Set 2.200_rev mascPCR	CAGCAATATTTGCCACCGCA
ybjK_rev	Set 2.150_rev mascPCR	AACTTTTCCGCAGGGCATC
rimK_rev	Set 2.100_rev mascPCR	TACAACCTCTTTCGATAAAAAGACCG
ygfJ_rev	Set 3.850_rev mascPCR	GATGAACTGTTGCATCGGCG
recJ_rev	Set 3.700_rev mascPCR	CTGTACGCAGCCAGCC
argO_rev	Set 3.600_rev mascPCR	AATCGCTGCCTTACGCG
yggU_rev	Set 3.500_rev mascPCR	TAACCAAAGCCACCAGTGC
mutY_rev	Set 3.400_rev mascPCR	CGCGAGATATTTTTTCATCATTCCG
glcC_rev	Set 3.300_rev mascPCR	GGGCAAAATTGCTGTGGC
yghQ_rev	Set 3.250_rev mascPCR	ACCAACTGGCGATGTTATTCAC
yghT_rev	Set 3.200_rev mascPCR	GACGATGGTGGTGGACGG
ygiZ_rev	Set 3.150_rev mascPCR	ATCGCCAAATTGCATGGCA
yqiB_rev	Set 3.100_rev mascPCR	AAAATCCTGACTCTGGCCTCA
endA.KO*	endA inactivating oligo	T*C*G*T*TTTAACACGGAGTAAGTGATGTACCGTTATTTGTCTATTGCTGCTGAGTGGTACTGAGCGCAGCATTTTCCGGCCCGGCGTTGGCC
exoX.KO*	exoX inactivating oligo	T*T*C*G*GCCTGGAGCATGCCATGTTGCGCATTATCGATACAGAAACTGATGCGGTTTGCAGGGAGGGATCGTTGAGATTGCCTCTGTTGATG
xseA.KO*	xseA inactivating oligo	G*A*A*T*TTGATCTCGCTCACATGTTACCTTCTCAATCCCCTGCAATTGATTTACCGTTAGTCGCCTGAATCAAACGGTTCGTCTGCTGCTTG
recJ.KO*	recJ inactivating oligo	G*G*A*G*GCAATTCAGCGGGCAAGTCTGCCGTTTCATCGACTTCACGTCACGACGAAGTTGTATCTGTTGTTTCACGCGAATTATTTACCGCT
xonA.KO*	xonA inactivating oligo	A*A*T*A*ACGGATTTAACCTAATGATGAATGACGGTAAGCAACAATCTGAACCTTTTTGTTTCACGATTACGAAACCTTTGGCACGCACCCCG

Lexo.KO.MM*	Lambda Exo inactivating oligo	T*G*A*A*ACAGAAAGCCGCAGAGCAGAAGGTGGCAGCATGACACCGTAACATTATCCTGCAGCGTACCGGGATCGATGTGAGAGCTGTCGAAC
endA.KO*-wt-f	endA wt-f mascPCR primer	CCGTTATTTGTCTATTGCTGCGG
exoX.KO*-wt-f	exoX wt-f mascPCR primer	GCGCATTATCGATACAGAAACCT
xseA.KO*-wt-f	xseA wt-f mascPCR primer	CTTCTCAATCCCCTGCAATTTTTACC
recJ.KO*-wt-f	recJ wt-f mascPCR primer	CAACAGATACAACTTCGTCGCC
xonA.KO*-wt-f	xonA wt-f mascPCR primer	GAATGACGGTAAGCAACAATCTACC
Lexo_WT-f	Lambda Exo wt-f mascPCR primer	GGCAGCATGACACCGGA
endA.KO*-mut-f	endA mut-f mascPCR primer	CCGTTATTTGTCTATTGCTGCTGA
exoX.KO*-mut-f	exoX mut-f mascPCR primer	GCGCATTATCGATACAGAAACTGA
xseA.KO*-mut-f	xseA mut-f mascPCR primer	CTTCTCAATCCCCTGCAATTGA
recJ.KO*-mut-f	recJ mut-f mascPCR primer	CAACAGATACAACTTCGTCGTGA
xonA.KO*-mut-f	xonA mut-f mascPCR primer	GAATGACGGTAAGCAACAATCTGA
Lexo_MUT-f	Lambda Exo mut-f mascPCR primer	TGGCAGCATGACACCGTAA
endA.KO*-r	endA rev mascPCR primer	GCACGATTGCAGATCAACAACG
exoX.KO*-r	exoX rev mascPCR primer	GACCATGGCTTCGGTGATG
xseA.KO*-r	xseA rev mascPCR primer	GGTACGCTTAAGTTGATTTTCCAGC
recJ.KO*-r	recJ rev mascPCR primer	GGCCTGATCGACCACTTCC
xonA.KO*-r	xonA rev mascPCR primer	GAAATGTCTCCTGCCAAATCCAC
Lexo-r	Lambda Exo rev mascPCR primer	CAAGGCCGTTGCCGTC
cat_mut*	cat inactivation oligo	G*C*ATCGTAAAGAACATTTTGAGGCATTTCAGTCAGTTGCTTAATGTACCTATAACCAGACCGTTCAGCTGGATATTACGGCCTTTTTA*A*A
cat_restore*	cat reactivation oligo (for coselection)	G*C*ATCGTAAAGAACATTTTGAGGCATTTCAGTCAGTTGCTCAATGTACCTATAACCAGACCGTTCAGCTGGATATTACGGCCTTTTTA*A*A
tolC-r_null_mut*	tolC inactivation oligo	A*G*CAAGCACGCCTTAGTAACCCGGAATTGCGTAAGTCTGCCGCTAAATCGTGATGCTGCCTTTGAAAAAATTAATGAAGCGCGCAGTCCA

tolC-r_null_revert*	tolC reactivation oligo (for coselection)	C*A*GCAAGCACGCCTTAGTAACCCGGAATTGCGTAAG TCTGCCGCCGATCGTGATGCTGCCTTTGAAAAAATTA ATGAAGCGCGCAGTCCA
bla_mut*	bla inactivation oligo	G*C*C*A*CATAGCAGAACTTTAAAAGTGCTCATCATTG GAAAACGTTATTAGGGGCGAAAACTCTCAAGGATCTT ACCGCTGTTGAGATCCAG
bla_restore*	bla reactivation oligo (for coselection)	G*C*C*A*CATAGCAGAACTTTAAAAGTGCTCATCATTG GAAAACGTTCTTCGGGGCGAAAACTCTCAAGGATCTT ACCGCTGTTGAGATCCAG
L:K.mut7-8.f	Forward primer for lacZ::kanR with distal mutations	TGACCATCCTTACGGATTCACTG
L:K.mut7-8.r	Reverse primer for lacZ::kanR with distal mutations	GTGCTGCTTGGCGATTAAG
L:K.mut7-8.L1-mut	mut mascPCR primer for lacZ::kanR 5' distal mutation	AGGAAACAGCTATGACCATCC
L:K.mut7-8.L1-wt	wt mascPCR primer for lacZ::kanR 5' distal mutation	CAGGAAACAGCTATGACCATGA
L:K.mut7-8.L4-mut	mut mascPCR primer for lacZ::kanR 3' distal mutation	CGTTACCCAACTTAATCGCCAA
L:K.mut7-8.L4-wt	wt mascPCR primer for lacZ::kanR 3' distal mutation	CGTTACCCAACTTAATCGCCTT
Kan.L1.rev	rev mascPCR primer for lacZ::kanR 5' distal mutation	ATGCATTTCTTTCCAGACTTGTTCA
Kan.L4.rev	rev mascPCR primer for lacZ::kanR 3' distal mutation	AGGGGACGACGACAGTATC
Kan:LacZ.NoPT-f	Forward primer for VPT1, VPT3, VPT6	TGACCATGATTACGGATTCACTGGCCGTCGTTTTACA A
Kan:LacZ.NoPT-r	Reverse primer for VPT1, VPT2, VPT5	GTGCTGCAAGGCGATTAAGTTGGGTAACGCCAGGGT TTTCCCAGT
Kan:LacZ.BlockPT-f	Forward primer for VPT5, VPT7	TGACCATGATTACGGATTCACTGGCCGTCGTTTTACA ACGT*C*G*T*G
Kan:LacZ.BlockPT-r	Reverse primer for VPT6, VPT7	GTGCTGCAAGGCGATTAAGTTGGGTAACGCCAGGGT TTTCC*C*A*G*T
Kan:LacZ.StartPT-f	Forward primer for VPT2, VPT4	T*G*A*C*CATGATTACGGATTCACTGGCCGTCGTTTTA CAA
Kan:LacZ.StartPT-r	Reverse primer for VPT3, VPT4	G*T*G*C*TGCAAGGCGATTAAGTTGGGTAACGCCAGG GTTTTCCCAGT
lacZ.seq-f	Forward primer for amplifying/sequencing	CGCAATTAATGTGAGTTAGCTCACTC

	lacZ ORF	
lacZ.seq-2-r	Reverse primer for amplifying/sequencing lacZ ORF	CGCCGAGTTAACGCCATCAA
LacZ7Stop.PT	Oligo for disrupting lacZ gene w/7 stop codons	T*A*G*C*GCAGCCTGAATGGCGAATAGCGCTTTGCCTAGTTTCCGGCACCATAAGCGGTGCCGTAAAGCTGGCTGTAGTGCGATCTTCC*T*T*A*G
