# Supplementary material for: Improving Lambda Red Genome Engineering in Escherichia coli via Rational Removal of Endogenous Nucleases
Source: PLoS One. 2012 Sep 5;7(9):e44638. doi: 10.1371/journal.pone.0044638 (PMC3434165; doi:10.1371/journal.pone.0044638)
Supplement: Table S2 — Recombination Frequencies of the VPT Cassette Series in Nuclease Knockout Strains. (PDF) [file pone.0044638.s002.pdf]

Table S2. Recombination Frequencies of the VPT Cassette Series in Nuclease Knockout Strains

This table reports the insertion frequencies of all seven VPT cassettes in the tested strains (EcNR2, nuc4$^-$, and EcNR2.*xseA*$^-$). Insertion frequencies were measured as the number of kanamycin resistant recombinants over the total number of cfu (as plated on non-selective media). These experiments were carried out in multiple replicates, as indicated.

VPT Series	EcNR2		Nuc4$^-$		EcNR2.*xseA*$^-$	
	Avg.	*St. Dev* (n=3)	Avg.	*Range* (n=2)	Avg.	*St. Dev* (n=3)
VPT1	4.67E-4	*2.38E-4*	2.32E-5	*1.27E-5 – 3.37E-5*	1.20E-3	*8.18E-5*
VPT2	1.73E-3	*6.27E-4*	3.08E-5	*1.94E-5 – 4.22E-5*	2.83E-3	*9.84E-4*
VPT3	1.11E-4	*3.03E-5*	4.29E-6	*4.01E-6 – 4.57E-6*	9.03E-5	*2.98E-5*
VPT4	5.97E-4	*5.01E-4*	1.72E-6	*1.68E-6 – 1.76E-6*	8.77E-5	*4.35E-6*
VPT5	1.26E-3	*9.75E-4*	1.42E-5	*1.28E-5 – 1.57E-5*	1.20E-3	*6.11E-4*
VPT6	2.28E-5	*1.93E-5*	3.04E-6	*2.91E-6 – 3.17E-6*	6.36E-5	*2.55E-5*
VPT7	4.60E-5	*3.04E-5*	2.44E-6	*1.80E-6 – 3.09E-6*	7.53E-5	*4.59E-5*
